# The sleep justice study - a prospective cohort study assessing sleep as a cardiometabolic risk factor after incarceration: a protocol paper

**DOI:** 10.1186/s12889-023-16985-x

**Published:** 2023-10-27

**Authors:** Johanna E. Elumn, Gul Jana Saeed, Jenerius Aminawung, Nadine Horton, Hsiu‑Ju Lin, H. Klar Yaggi, Emily A. Wang

**Affiliations:** 1 SEICHE Center for Health and Justice, Yale School of Medicine, New Haven, CT USA; 2 Department of Internal Medicine, Section of General Internal Medicine, Yale School of Medicine, New Haven, CT USA; 3 Department of Internal Medicine, Roger Williams Medical Center, Providence, RI USA; 4 Department of Social Work, University of Connecticut, Storrs, CT USA; 5 Section Pulmonary, Critical Care and Sleep Medicine, Department of Internal Medicine, Yale School of Medicine, New Haven, CT USA; 6 VA CT Clinical Epidemiology Research Center (CERC), West Haven, CT USA

**Keywords:** Incarceration, Sleep, Cardiovascular risk, Prospective cohort

## Abstract

**Background:**

An estimated 11 million individuals are released from U.S. jails and prisons each year. Individuals with a history of incarceration have higher rates of cardiovascular disease (CVD) events and mortality compared to the general population, especially in the weeks following release from carceral facilities. Healthy sleep, associated with cardiovascular health, is an underexplored factor in the epidemiology of CVD in this population. Incarcerated people may have unique individual, environmental, and institutional policy-level reasons for being sleep deficient. The social and physical environment within carceral facilities and post-release housing may synergistically affect sleep, creating disparities in sleep and cardiovascular health. Since carceral facilities disproportionately house poor and minoritized groups, population-specific risk factors that impact sleep may also contribute to inequities in cardiovascular outcomes.

**Methods:**

This study is ancillary to an ongoing prospective cohort recruiting 500 individuals with known cardiovascular risk factors within three months of release from incarceration, the Justice-Involved Individuals Cardiovascular Disease Epidemiology (JUSTICE) study. The Sleep Justice study will measure sleep health among participants at baseline and six months using three validated surveys: the Pittsburgh Sleep Quality Index (PSQI), the STOP-Bang, and the Brief Index of Sleep Control. In a subsample of 100 individuals, we will assess sleep over the course of one week using wrist actigraphy, a validated objective measure of sleep that collects data on rest-activity patterns, sleep, and ambient light levels. Using this data, we will estimate and compare sleep health and its association with CVD risk factor control in individuals recently released from carceral facilities.

**Discussion:**

The incarceration of millions of poor and minoritized groups presents an urgent need to understand how incarceration affects CVD epidemiology. This study will improve our understanding of sleep health among people released from carceral facilities and its potential relationship to CVD risk factor control. Using subjective and objective measures of sleep will allow us to identify unique targets to improve sleep health and mitigate cardiovascular risk in an otherwise understudied population.

## Background

Sleep health is a multidimensional construct with overlapping components: duration, timing, regularity, efficiency, satisfaction, and impact on daytime alertness [1, 2]. Prior research has identified poor sleep as a risk factor for all-cause mortality [3] and has explored potential mechanisms implicating cardiometabolic health [4, 5]. Recent American Heart Association guidelines updates have incorporated sleep as an essential eighth pillar of ideal cardiovascular health (AHA Life’s Essential 8) [6]. Suboptimal sleep can contribute to hypertension, obesity, and diabetes and is also associated with cardiovascular disease (CVD) [3, 7–9], and sleep health disparities may contribute to CVD [10]. While environmental factors, including sleep environment, housing, and neighborhood characteristics, contribute to sleep health and CVD risk factor management, exposure to the carceral facility environment remains an area of limited research [11, 12].

The United States incarcerates more people per capita than any other country in the world [13], with over 2 million people incarcerated and another 5 million facing parole or probation—time served under community supervision instead of in a carceral facility [13]. At any given time, an estimated 2.7% of individuals residing in the United States have a history of incarceration [14, 15]. Significant disparities exist in those more likely to be incarcerated, partly due to structural racism and other social determinants of health [16, 17]. Minoritized groups are disproportionately incarcerated, with Black people incarcerated at five times and Hispanic people at 2.5 times the rate of White people [18, 19]. While there are documented sleep health disparities among minoritized racial and ethnic groups, incarceration is an unexplored factor contributing to these disparities. Prior studies have shown that exposure to incarceration ranging from being incarcerated, [20–22] to having a family member incarcerated [23], and even living in a neighborhood with high incarceration rates is associated with worse CVD outcomes. CVD is a major reason for hospitalization among people with a history of incarceration and is a leading cause of death during incarceration and after release [24–26]. The factors elevating CVD risk in this population are largely unknown and only partly driven by a higher prevalence of conditions and risk factors associated with heart diseases, such as smoking [27, 28], diabetes, hypertension, and obesity [28, 29].

There remains a dearth of existing literature on sleep health as a potential CVD mediator. Incarcerated people have little control over their sleep health. Exposure to noise and light, uncomfortable sleeping conditions, limited opportunities for physical activity, boredom, and loss of autonomy can all contribute to decreased sleep quality, including under-sleeping, oversleeping, and sleep disturbance [30–32]. Furthermore, incarcerated people are more likely to witness and directly experience violence and have physical or mental health conditions, which can, in turn, affect sleep health by causing nightmares, insomnia, sleep apnea, and general sleep disruption. Sleep disruption associated with trauma exposure often lingers after other trauma symptoms have resolved, putting individuals at continued elevated risk for CVD [33, 34]. Post-traumatic stress disorder (PTSD) can also contribute to CVD through other pathways, including worse management of CVD risk factors (e.g., diabetes, hyperlipidemia, hypertension) [35–38]. People recently released from carceral facilities also experience significant stress from housing and economic insecurity and family instability, factors that potentially impact sleep health [39–42]. The relationship between sleep disorders and CVD risk factor control and the role of PTSD as a potential mediator remains unexplored in populations involved in the criminal legal system. The few studies on sleep health among people in correctional facilities focus primarily on insomnia [31, 43–46], and sleep health among formerly incarcerated individuals remains an area of limited research.

In our study, we will explore various aspects of sleep health, including quality, duration, disturbance, efficiency, satisfaction, sleep-wake patterns, and the impact of sleep on daily functioning, after release from carceral facilities and whether sleep problems persist six months after release. Because a short-term stay in a carceral facility may affect sleep differently than a longer sentence, we will also examine differences in sleep health and the length of the participant’s last incarceration.

This study aims to understand how sleep health is associated with CVD risk factor control among individuals released from carceral facilities and the potential role of PTSD in the relationship between sleep health and CVD risk.

## Methods

### Study objectives

The Sleep Justice study is designed to understand how sleep health is associated with CVD risk factor control among individuals released from carceral facilities and the role of PTSD as a potential mediating factor. To achieve this, we built upon the infrastructure of an ongoing R01, JUSTice Involved Individuals Study of Cardiovascular Disease Epidemiology (JUSTICE) study [47] and added subjective and objective sleep measures to the ongoing cohort study. In addition, this study of sleep after release from incarceration adds a novel exploration of the role of sleep and PTSD in CVD risk factor control. To our knowledge, this is the first prospective study to examine sleep after release from a carceral facility.

### Study design

#### Participants

We plan to recruit and enroll 300 participants in the Sleep Justice study. The study inclusion criteria require that participants [1] were released from jail or prison in the last 3 months, [2] have CVD or a CVD risk factor at baseline, and [3] be released to Bridgeport, Hartford, or New Haven, Connecticut. Recruitment will occur in the community and through outreach to halfway houses, reentry programs, probation, and parole.

#### Data collection and measurements

We will collect both objective and subjective sleep data on the various dimensions of sleep health using the measures described here. Sleep health includes several dimensions of sleep, including duration, efficiency, timing, alertness, and satisfaction/quality [2]. By collecting subjective and objective sleep data in a sample of people recently released from a carceral facility, we can examine this data in relation to their cardiovascular disease risk factors and personal demographic data. We will collect data on [1] sleep (subjective and objective), [2] health conditions, [3] access to care for sleep conditions during incarceration, [4] PTSD, [5] current sleep environment, and [6] clinical measures of CVD risk factors.

Data collection for Sleep Justice began in November 2020 and will continue until the target sample for the study has been completed. We estimate that we will administer the subjective measures to 300 participants during the study period and the objective measurement of sleep to 100 participants (Table 1). Subjective sleep data will be collected in the baseline survey along with additional measures (SEE JUSTICE protocol) [47] by administering the Pittsburgh Sleep Quality Index (PSQI) [48], the STOP-Bang [49], and the Brief Index of Sleep Control (BRISC) [50]. The PSQI was designed to assess sleep quality over one month and includes subscores that capture sleep disturbances, duration, efficiency, latency, daytime dysfunction, and use of medications for sleep. We included the STOP-Bang as a screening tool for sleep apnea because sleep apnea contributes to CVD risk [49]. The BRISC assesses the amount of control the person has over their sleep and is scored to indicate a range of control over their sleep from no control to complete control. The BRISC was added because this population may have less control over their sleep than others, and this aspect of sleep may contribute to sleep health disparities [51–53]. We will supplement the subjective data with objective sleep data from a subsample of 100 participants using wrist actigraphy. Using wrist actigraphy, we can longitudinally measure participants’ sleep and activity in and out of home environments with limited inconvenience to the wearer [54].

**Table 1:** Sleep Justice data collection

Domain	Components/method of assessment	When Assessed	Purpose
**Demographic**	Age, sex, race, ethnicity, level of education	BL	Covariate
**Clinical**			
Blood pressure	Physical examination	BL, 6 and 12 months after release	Outcome
Height	Physical examination	BL, 6 and 12 months after release	Outcome
Weight	Physical examination	BL, 6 and 12 months after release	Outcome
Neck circumference	Physical examination	BL, 6 and 12 months after release	Outcome
Lipid panel	Point of care blood test	BL, 6 and 12 months after release	Outcome
Glycosylated hemoglobin (HbA1c)	Point of care blood test	BL, 6 and 12 months after release	Outcome
**Psychosocial factors**			
Post-traumatic stress disorder	PTSD symptom scale [55]	BL, 6 and 12 months after release	Covariate
**Sleep**			
Sleep quality	Pittsburgh Sleep Quality Index (PSQI) [48]	BL, 6 and 12 months after release	Predictor
Sleep apnea risk	Stop-Bang [49]	BL	
Sleep control	Brief Index of Sleep Control [50]	BL, 6 and 12 months after release	
Objective sleep data	Actiwatch Spectrum Plus	BL	Predictor

*BL* Baseline

We decided to collect objective wrist actigraphy sleep data [56] with the Actiwatch Spectrum Plus because many participants recently released from a carceral facility do not have cell phones. We will collect seven days of sleep data using the Actiwatch: rest-activity patterns, objective sleep data (e.g., habitual sleep/wake time, sleep efficiency), and the amount of light in the sleep environment. The Actiwatch will be distributed to this subsample of participants at the baseline study visit. In addition, we ask participants to complete a sleep diary, take a photo of the place where they sleep, and then return all three to the research assistant after one week. We will assess the feasibility and acceptability of using wrist actigraphy, completing the sleep diary, and submitting a photo of the sleep space.

We will also use data collected through the parent JUSTICE study survey which includes information on demographics, psychosocial/behavioral/medical factors, and experiences of carceral and incarceration-related post-release policies. Information on PTSD will be collected using the PTSD symptom scale. The study’s clinical assessment includes height, weight, neck circumference, blood pressure, and point-of-care testing (lipid panel, glycosylated hemoglobin (HbA1c), and urine toxicology screen). Participants are paid $60 for completing the baseline and 6-month survey and clinical assessments and $50 for wearing the Actiwatch Spectrum Plus for 7 days and returning it for data download along with the completed sleep diary and sleep space photo.

### Follow-up and retention

At baseline, we collect participant names, aliases, phone numbers, addresses, dates of birth, social security numbers, inmate numbers, and Medicaid numbers for study follow-up. In addition, to facilitate study retention and follow-up, we will also collect contact information for five members of their social network (family members or friends) who could locate them.

Participants are contacted by phone or text two weeks before the follow-up survey to remind them of their upcoming survey and clinical assessment. After making three attempts to contact participants, we will use information from study locator forms, a search of the Department of Corrections inmate locator system, and contacts they provide at the baseline visit. In between the baseline and follow-up visits, participants are paid $5 for phone check-ins and $20 for phone interviews. Participants who are re-incarcerated during the study can complete the survey by mail, and incentives for their participation can be held until their release or given to a designated family member to follow the CT DOC rules that prevent research compensation during incarceration.

### Analytic approach

We will examine the association between sleep health and CVD risk factor control using sleep and clinical data from the survey. We also included questions about access to sleep disorder treatment in carceral facilities, including access to medication and the use of continuous positive airway pressure (CPAP) machines, to assess whether those diagnosed with obstructive sleep apnea received treatment during incarceration and potential differences in access by type of facility (e.g., jail vs. prison). We will use both subjective and objective data to understand sleep health in this population. We will operationalize and evaluate sleep health as [1] duration (measured by Actiwatch/PSQI/sleep diary), [2] efficiency (Actiwatch/PSQI), [3] timing (Actiwatch/PSQI/sleep diary), [4] alertness (PSQI), [5] satisfaction (PSQI), [6] sleep apnea risk (STOP-Bang), [7] sleep control (BRISC). In the analysis, we will look for correlations between the dimensions of sleep health. We will create a sleep health score that is the sum of each of the dimensions derived from the data collected based on a review of the existing literature. We will then examine how changes in sleep health affect CVD risk factor control by using sleep measurement data collected at baseline and the 6-month follow-up survey and whether screening positive for PTSD (measured by the PTSD symptom scale) mediates the relationship between sleep and CVD risk factor control.

#### Statistics strategy and planned analysis

We will employ Generalized Estimating Equations (GEE) to evaluate the association between sleep health, other psychosocial stress measures (i.e., PTSD), and CVD risk factor control [57]. We have chosen GEE as it handles repeated continuous or categorical responses that are clustered and correlated and accommodates unbalanced data resulting from attrition during follow-up measures, meaning that having an equal number of repeated measures for each individual is unnecessary. In other words, all study participants, including those with missing follow-up data, will be included in the analyses. The primary predictor will be the PSQI score at the six-month follow-up. The primary outcome will be a single measure of any uncontrolled CVD risk factor at baseline and 6 months. Then we will examine secondary outcomes for each risk factor SBP ≥ 140 or DBP ≥ 90, BMI ≥ 30, A1c ≥ 8, or LDL ≥ 160. The sample size was estimated to detect a small effect size at 80% of statistical power.

#### Sample size statistical power estimation

This study will recruit 300 participants for the subjective sleep measures, incarceration history, health, and clinical assessment data and a subsample of 100 for the objective sleep measurement using wrist actigraphy. We will focus our power analysis on the minimum detectable effect size (MDES) [58]. The MDES represents the smallest effect that a study can detect given a predetermined number of participants and the level of statistical power. A smaller MDES indicates higher precision, meaning that a study with high precision can detect even the slightest effect size. To estimate the detectable effect size, we used the algorithm developed by Li and McKeague [59, 60]. Given the proposed 300 that we expect to recruit for this study, and assuming a significance level of 0.05 (two-tailed test), an intra-cluster correlation of 0.5 (which is consistent with several of the related measures and the literature, see Gibbons et al.) [61], and 80% of power, the MDES as indicated by odds ratio is 1.67, which is equivalent to equivalent to a small effect size, between sleep disturbance and any uncontrolled CVD risk factor [62]. For the objective sleep measurement, we proposed to recruit 100 participants for actigraphy assessment and compare these results with the responses to the sleep measures. We will look at correlations between the actigraphy data and the PSQI scores at baseline. Given this sample size (assuming 80% statistical power, two-sided test with a significance level of 0.05), we will only be able to detect a medium-small effect size of OR = 1.96 (*n* = 100).

#### Ethics

This study was approved by the institutional review board of Yale University (HIC #2,000,022,213) and Connecticut Department of Corrections (CT DOC) Research Advisory Committee. A Certificate of Confidentiality protects study data. Ensuring participant privacy and confidentiality in the conduct of this study are paramount to the research team and were considered in the collection, storage, analysis, and dissemination of study data. Our research approach of ensuring the inclusion of people with a history of incarceration in the research, from design to dissemination of the study, also complements the standard protection of human subjects practices.

#### Dissemination plan

Study results will also be disseminated to study participants, incarcerated and formerly incarcerated people, carceral systems, and community partners who work with this population through a variety of methods, including, but not limited to, community presentations at local halfway houses, reentry organizations, and community meetings, reports, and through our website and social media. They will also disseminate the results through academic journals to reach other researchers focused on incarceration, sleep, and health.

## Discussion

Our study will contribute knowledge on the sleep health of people released from carceral facilities. It will help identify factors associated with poor sleep and cardiovascular health outcomes in this population. By measuring incarceration-related exposures, psychosocial factors, subjective and objective measures of sleep, and their relation to overall cardiovascular risk, we hope to identify unique targets for intervention to improve care for and mitigate cardiovascular risk in the millions of individuals released from incarceration each year. A 6-month longitudinal follow-up will also allow us to assess how these risk factors evolve over time. This study will also address the paucity of sleep research on how incarceration in U.S. carceral facilities impacts sleep during incarceration and after release. These results will help inform public health policymakers, carceral systems, and reentry service providers regarding potential population and individual-level interventions, which in turn help reduce the burden of cardiovascular disease and improve health outcomes due to poor sleep in this often overlooked population.

## Data Availability

In compliance with NIH policy, we will make the study results available to study participants, the general public, and researchers. After the publication of the main study findings, the final deidentified dataset will be made available to other researchers upon request per NIH guidelines. Details of the study and dataset, including information about the methodology, data collection, and the data dictionary, will also be provided along with the final dataset.

## Abbreviations

BRISC: Brief Index of Sleep Control
CVD: Cardiovascular disease
CT DOC: Connecticut Department of Corrections
GEE: Generalized Estimating Equations
JUSTICE: JUSTice Involved Individuals Study of Cardiovascular Disease Epidemiology
MDES: Minimum Detectable Effect Size
PSQI: Pittsburgh Sleep Quality Index
PTSD: Post-traumatic stress disorder
